# Temporal trends in incidence and mortality from pulmonary tuberculosis: time series study, Sul da Bahia, 2010-2023

**DOI:** 10.1590/S2237-96222025v34e20240778.en

**Published:** 2025-08-04

**Authors:** Marília Caixeta de Araujo, Renata Soares Passinho, Renan Sallazar Ferreira Pereira, Delio José Mora

**Affiliations:** 1 Universidade Federal do Sul da Bahia, Centro de Formação em Ciências da Saúde, Teixeira de Freitas, BA, Brasil; 2 Universidade Federal de São João del-Rei, Departamento de Enfermagem, Divinópolis, MG, Brazil

**Keywords:** Pulmonary Tuberculosis, Epidemiological Monitoring, Communicable Diseases Control, Health Information Systems, Time Series Studies, Tuberculosis Pulmonar, Monitoreo Epidemiológico, Control de Enfermedades Transmisibles, Sistemas de Información de Salud, Etudios de Series de Temporales

## Abstract

**Objective:**

To analyze the temporal trend of incidence and mortality from pulmonary tuberculosis in Southern Bahia.

**Methods:**

Time series study of new cases and deaths from pulmonary tuberculosis conduccted on Costa do Descobrimento and in the Extremo Sul of Bahia. The number of new cases and deaths from pulmonary tuberculosis was obtained from the Notifiable Diseases Information System, between 2010 and 2023, and used to calculate incidence and mortality. Prais-Winsten regression was used to calculate the annual percentage variation (APV) and 95% confidence interval (95%CI) and to classify trends.

**Results:**

4,005 new cases and 128 deaths from pulmonary tuberculosis were registrated during the period of the study. The average incidence was 34.91 cases per 100,000 inhabitants, and mortality was 1.11 deaths per 100,000 inhabitants. Incidence and mortality showed stationary trends. Decreasing incidence occurred in females (APV -0.01; 95%CI -0.02; -0.01) and in the age groups of 0-9 years (APV -0.02; 95%CI -0.04; -0.01), 40-59 years (APV -0.01; 95%CI -0.02; -0.01) and 60 years or older (APV -0.01; 95%CI -0.04; -0.01). Costa do Descobrimento showed a decreasing incidence for females (APV -0.02; 95%CI -0.03; -0.01) and for the age group of 40-59 years (APV -0.02; 95%CI -0.03; -0.01). In Extremo Sul, the incidence decreased in the 20-39 age group (APC -0.01; 95%CI -0.02; -0.01).

**Conclusion:**

Although temporal trend of incidence and mortality were stationary, public health strategies are necessary, especially on Costa do Descobrimento, which maintained the highest rates of pulmonary tuberculosis.

## Resumen

**Objetivo:**

Analizar la tendencia temporal de la incidencia y mortalidad por tuberculosis pulmonar en el sur de Bahía.

**Métodos:**

Estudio de series temporales de casos nuevos y muertes por tuberculosis pulmonar en la Costa do Descobrimento y en el Extremo Sur de Bahía. El número de casos nuevos y muertes por tuberculosis pulmonar se obtuvo del Sistema de Registro y Notificación de Incidentes, entre 2010 y 2023, y se utilizó para calcular la incidencia y la mortalidad. Se utilizó la regresión de Prais-Winsten para calcular el cambio porcentual anual (VPA) y el intervalo de confianza del 95% (IC95%) y clasificar las tendencias.

**Resultados:**

Se registraron 4.005 casos nuevos y 128 fallecimientos por tuberculosis pulmonar. La incidencia media fue de 34,91 casos por cada 100.000 habitantes y la mortalidad de 1,11 muertes por cada 100.000 habitantes. La incidencia y la mortalidad mostraron tendencias estacionarias. Se observó una incidencia decreciente en mujeres (APC -0,01; IC95% -0,02; -0,01) y en los grupos de edad de 0 a 9 años (APC -0,02; IC95% -0,04; -0,01), 40 a 59 años (APC -0,01; IC95% -0,02; -0,01) y 60 años o más (APC -0,01; IC95% -0,04; -0,01). La Costa del Descubrimiento mostró una incidencia decreciente para las mujeres (VPA -0,02; IC del 95% -0,03; -0,01) y el grupo de edad de 40 a 59 años (VPA -0,02; IC del 95% -0,03; -0,01). En el extremo sur, la incidencia disminuyó en el grupo de edad de 20 a 39 años (APC -0,01; IC95% -0,02; -0,01).

**Conclusión:**

Aunque la tendencia temporal de la incidencia y la mortalidad ha sido estacionaria, son necesarias estrategias de salud pública, especialmente en la Costa do Descobrimento, que mantuvo las tasas más altas de tuberculosis pulmonar.

**Ethical aspects**

This research used public domain data and anonymized databases.:

## Introduction

Tuberculosis is a neglected disease that mainly affects populations with greater socioeconomic vulnerability (1). It is estimated that one third of the world’s population is infected with *Mycobacterium tuberculosis* and that annualy the disease affects 7.5 million people and causes 1.3 million deaths (1.2). In 2023, Brazil registered 80,012 new cases of tuberculosis, of which 88.0% were pulmonary, with an incidence of 32.75 pulmonary cases/100,000 inhabitants (3). 

A decline in tuberculosis incidence in Brazil for all age groups was observed from 2001 to 2017 (4.5). Tuberculosis incidence rates were higher in males, except in the 10-14 age group (3). The decreasing trend in deaths in the country was observed between 1990 and 2015, and individuals under 15 years of age recorded a reduction in mortality between 1996 and 2020, with the greatest drop for the 0-4 age group (6,7).

In Brazil, social inequalities reflect the heterogeneity in tuberculosis indicators (4-6). In 2023, Bahia had an incidence of 25.90 cases/100,000 inhabitants and the worst performance in the Northeast in terms of recovery cases (3). Between 2001 and 2017, Bahia recorded a drop in the incidence of tuberculosis for all age groups and, between 1990 and 2015, the trend of deaths in the state was decreasing (3,6,7). 

The Brazilian Unified Health System provides access to diagnosis and treatment for tuberculosis throughout the country, but these actions occur unevenly (8). Access to health services is associated to one’s social and residential conditions. This means that tuberculosis patients living in regions with less socioeconomic development face difficulties, which impacts the incidence and mortality from pulmonary tuberculosis (9). 

Identifying populations most exposed to tuberculosis allows planning prevention and control actions in areas of greatest risk (10). As there is a shortage of studies on tuberculosis in regions of Bahia and time series can provide a certain longitudinal and dynamic view of the population’s health (10), analysis from the point of view of sex and age group is relevant to assess which groups would be most affected by tuberculosis. This study sought to analyze the temporal trend of incidence and mortality from pulmonary tuberculosis in two territories in southern Bahia. 

## Methods

### Study design

This was a time series study aimed at analyzing the incidence and mortality from pulmonary tuberculosis in southern Bahia between 2010 and 2023.

### Context

**Figure 1:**
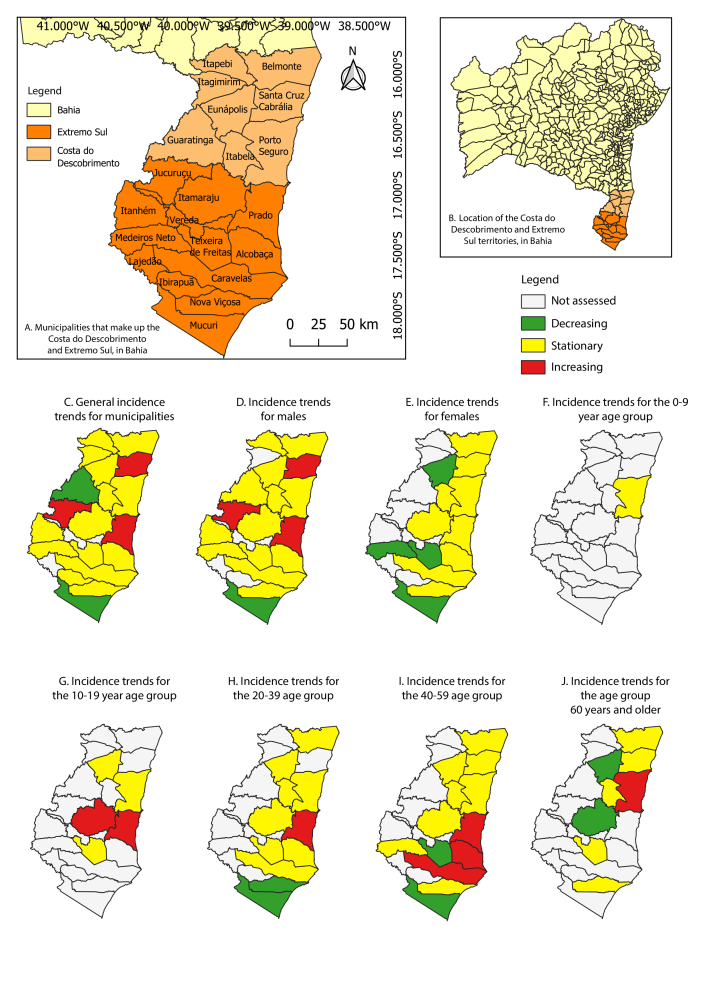
Costa do Descobrimento and Extremo Sul, in the state of Bahia, and classification of trends for incidence rates of pulmonary tuberculosis in their municipalities, 2010-2023 (n=4,005)

The Costa do Descobrimento and Extremo Sul territories are located in the south of Bahia (Figure 1A and 1B). Their combined population is 824,903 inhabitants, representing 5.8% of the population of Bahia. 

### Participants

The study population was composed of individuals included in the Notifiable Diseases Information System (SINAN) as a confirmed case of tuberculosis, with the “pulmonary” as clinical form and “new case” as the entry type. New cases were people with tuberculosis who did not use anti-tuberculosis medication or have used it for less than a month (3). 

### Variables

The selected data were: year of notification (2010-2023), sex (male, female) and age group in years (0-9, 10-19, 20-39, 40-59 and 60 or more). For the analysis of mortality, the number of new cases with the outcome “death from tuberculosis” was used. 

In calculating the incidence and mortality rates for pulmonary tuberculosis, the 2010 and 2022 Demographic Censuses and intercensal population estimates, made available by the Brazilian Institute of Geography and Statistics (11,12) were used. The number of new cases and deaths from new cases was divided by the resident population and multiplied by 100,000 inhabitants. The estimated population for each sex and age group category was used to calculate incidence and mortality rates in the territories and their municipalities.

### Data sources and measurement

The data were obtained from Sinan, made available by the Department of Information and Informatics of the Unified Health System and accessed via Tabnet in March 2024 (13). The path to access the data was (in Portuguese): Acesso à informação > Informações de saúde (Tabnet) > Epidemiológicas e morbidade > Casos de tuberculose – desde 2,001 > Abrangência geográfica Bahia. Then, the “ano de diagnóstico” (year of diagnosis) line, the “sexo” (sex) or “faixa etária” (age group) column and the “casos confirmados” (confirmed cases) content were selected. In the “seleções disponíveis” (available selections) tab, “Município de notificação” (Notification municipality) section, each of the 21 municipalities that make up the territories of interest was selected. In “seleções disponíveis” (available selections), the options “caso novo” (new case) in “Tipo de entrada” (Entry type) and “pulmonar” (pulmonary) in “Forma” (Form) were selected. For the mortality analysis, “óbito por tuberculose” (death from tuberculosis) was selected in “Situação de encerramento” (Outcome status).

Data on the population of municipalities were made available by the Brazilian Institute of Geography and Statistics and accessed in March 2024 via the Bahia Health Department (11-13). The path to access the data was (in Portuguese): Vigilância em saúde > Vigilância epidemiológica > Demografia > População residente > Estratificada por sexo e faixa etária. Then, the “ano” (year) line, the “sexo” (sex) or “faixa etária” (age group) column and the “população residente” (resident population) content were selected. In the “seleções disponíveis” (available selections) tab, the 21 municipalities investigated were selected in the “Município” (Municipality) section.

### Bias control

The data was correlated in time series, meaning that the value at one point in time was related to values at previous points. To correct this bias, the Prais-Winsten technique (10) was applied. 

Ecological bias was controlled by not assuming that associations observed at the aggregate level could be applied individually, generating incorrect conclusions about causal relationships. 

Information bias was related to the quality and accuracy of secondary data, as incomplete, inaccurate or poorly documented data can distort results. This bias was controlled by choosing the variables “sex” and “age group” that did not have the “unknown/blank” category.

### Statistical methods

The data were presented using central tendency and dispersion measures for quantitative variables and frequency measures for qualitative variables.

Prais-Winsten regression was used to analyze the time series of incidence and mortality rates. In the analysis, time (year) was considered an independent variable, and incidence and mortality rates were the dependent variable. The logarithmic transformation of the mentioned rates was performed to reduce the effect of data heterogeneity, correct deviations from normality and stabilize the variance over time (10).

Based on the regression, the annual percentage variation and 95% confidence intervals (95%CI) were estimated. The trend was increasing, if p-value<0.05 and positive annual percentage variation; decreasing, if p-value<0.05 and negative annual percentage variation; and stationary, if p-value≥0.05. Analyses were performed using STATA software version 14.0 (StataCorp LLC, College Station, TX, USA).

## Results

**Figure 2:**
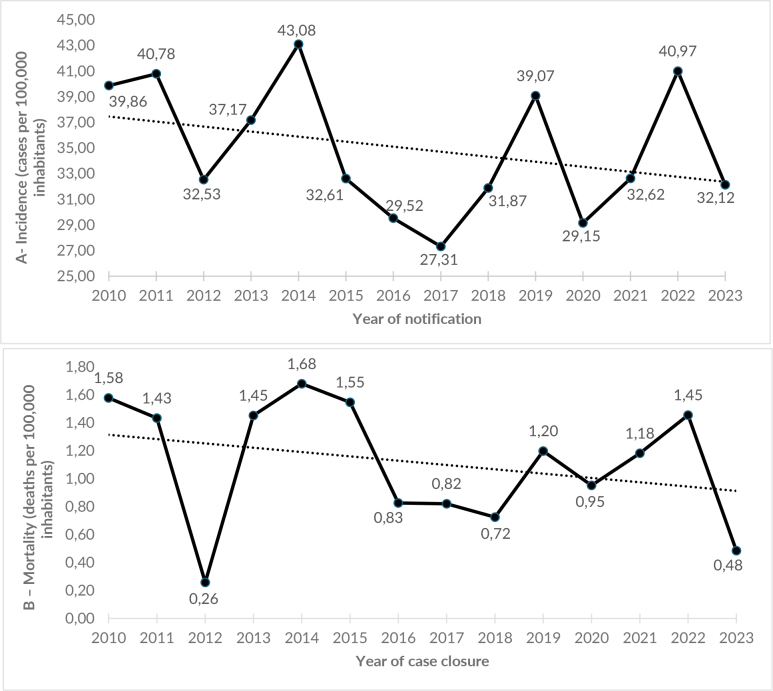
Time series of the incidence rate (A) and mortality rate (B) for pulmonary tuberculosis. Costa do Descobrimento and Extremo Sul, Bahia, 2010-2023 (n=4,005)

In the period 2010-2023, 4,005 new cases of pulmonary tuberculosis were reported, with an average incidence of 34.91 (±5.10) cases/100,000 inhabitants for the territories. The incidence distribution identified peaks of new cases in 2011, 2014, 2019 and 2022 and declines in 2017 and 2020 (Figure 2A). The highest number of new cases was recorded in males (67.0%) and in the 20-39 age group (43.0%). 

During the study period, 128 new case outcomes of death from pulmonary tuberculosis were observed. The highest number of deaths occurred among males (72.0%) and in the population aged 40-59 (37.0%). The global average was 1.11 (±0.44) deaths/100,000 inhabitants (Figure 2B).

The distribution of incidence and mortality rates was observed according to sex and age group (Table 1). The average incidence was higher in males and in the 40-59 age group in both territories. Average mortality was higher in males and in the age group of 60 years or older.

**Table 1:** Distribution of incidence and mortality rates of pulmonary tuberculosis, according to sex and age group. Costa do Descobrimento and Extremo Sul, Bahia, 2010-2023 (n=4,005)

Variable	2010	2011	2012	2013		2014	2015	2016	2017	2018	2019	2020	2021	2022	2023	Mean (standard deviation)
	Incidence rate (per 100,000 inhabitants)															
Gender																
Male	54.28	54.29	38.35		47.57	54.57	42.48	39.09	37.88	43.35	53.31	38.55	46.14	59.97	47.68	46.96 (±7.33)
Female	25.34	27.19	26.68		26.72	31.53	22.67	19.89	16.69	20.34	24.75	19.87	19.03	22.25	16.98	22.85 (±4.34)
**Age group** (years)																
0-9	7.24	3.58	4.97		4.66	6.60	6.54	3.25	3.87	3.32	2.64	3.04	1.51	4.10	3.28	4.1 9(±1.65)
10-19	15.78	14.33	11.62		15.14	15.61	10.71	8.86	13.50	9.07	13.81	16.40	10.62	19.07	18.31	13.77 (±3.23)
20-39	53.81	54.45	39.56		52.36	52.59	44.63	37.53	31.98	43.04	56.35	37.73	38.91	53.17	41.67	45.56 (±7.99)
40-59	60.29	69.69	58.54		51.83	85.03	50.96	51.17	44.85	56.06	63.00	40.18	49.64	55.15	46.04	55.89 (±11.38)
≥60	63.22	61.27	52.13		63.93	51.30	49.60	51.94	50.10	46.28	52.57	36.45	53.34	52.11	32.34	51.19 (±8.85)
	Mortality rate (per 100,000 inhabitants)															
Gender																
Male	2.36	2.08	0.51		2.41	1.91	1.42	1.18	1.64	1.45	1.91	1.44	1.66	1.47	0.98	1.60 (±0.52)
Female	0.79	0.78	0.00		0.49	1.44	1.67	0.47	0.00	0.00	0.48	0.47	0.70	1.44	0.00	0.62 (±0.56)
**Age group** (years)																
0-9	0.72	0.00	0.00		0.00	0.00	0.65	0.00	0.65	0.66	0.00	0.00	0.00	0.00	0.00	0.19 (±0.32)
10-19	0.00	0.00	0.00		0.00	0.60	0.00	0.59	0.00	0.00	0.00	0.00	0.00	0.00	0.76	0.14 (±0.28)
20-39	0.79	1.57	0.78		1.82	0.72	1.79	0.00	0.35	0.36	0.72	0.36	1.44	1.19	0.00	0.85 (±0.62)
40-59	2.68	1.99	0.00		1.85	3.67	1.82	1.20	1.79	2.46	1.84	3.09	1.02	3.36	0.48	1.95 (±1.06)
≥60	7.35	5.84	0.00		5.44	6.75	5.36	5.33	2.64	0.00	6.74	1.01	4.03	1.80	1.80	3.86 (±2.60)

For Costa do Descobrimento, the average incidence was 34.93 (±7.81) cases/100,000 inhabitants, and, for Extremo Sul, 26.73 (±4.62) cases/100,000 inhabitants. The incidence of pulmonary tuberculosis was higher on Costa do Descobrimento for both sexes and in all age groups when compared to Extremo Sul. For Costa do Descobrimento, the average mortality rate was 1.25 (±0.80) deaths/100,000 inhabitants, and, for Extremo Sul, 0.53 (±0.30) deaths/100,000 inhabitants. For females, average mortality was the same in both territories, while for males it was higher on Costa do Descobrimento. For the 0-9 age group, the same average mortality rate was observed for the territories. For the other age categories, mortality was higher on Costa do Descobrimento. Both territories had the highest average mortality for the age group of 60 years or older (Table 1). 

The incidence of pulmonary tuberculosis in both territories was stationary, with a decrease for females and the age groups 0-9, 40-59 and 60 years or older. On Costa do Descobrimento, the incidence reduced for females and in the 40-59 age group. In Extremo Sul, the incidence fell for the 20-39 age group (Table 2). 

**Table 2:** Annual percentage variation (APV), 95% confidence interval (95%CI) and interpretation of temporal trends in the incidence and mortality of general pulmonary tuberculosis, according to sex and age group. Costa do Descobrimento and Extremo Sul, Bahia, 2010-2023 (n=4,005)

	Incidence rate			Mortality rate		
Location	APV (95%CI)	p-value	Interpretation	APV (95%CI)	p-value	Interpretation
Territories	-0.01 (-0.01; 0.01)	0.278	Stationary	-0.01 (-0.02; 0.01)	0.423	Stationary
Gender						
Male	-0.01 (-0.01; 0.01)	0.912	Stationary	-0.01 (-0.01; 0.01)	0.332	Stationary
Female	-0.01 (-0.02; -0.01)	0.013	Decreasing	Not assessed	Not assessed	Not assessed
**Age group** (years)						
0-9	-0.02 (-0.04; -0.01)	0.007	Decreasing	Not assessed	Not assessed	Not assessed
10-19	0.01 (-0.01; 0.02)	0.594	Stationary	Not assessed	Not assessed	Not assessed
20-39	-0.01 (-0.02; 0.01)	0.295	Stationary	-0.01 (-0.03; 0.01)	0.154	Stationary
40-59	-0.01 (-0.01; -0.01)	0.014	Decreasing	0.01 (-0.02; 0.02)	0.762	Stationary
≥60	-0.01 (-0.02; -0.01)	<0.001	Decreasing	-0.02 (-0.06; 0.01)	0.214	Stationary
**Costa do Descobrimento**	-0.01 (-0.02; 0.01)	0.050	Stationary	-0.01 (-0.02; 0.01)	0.650	Stationary
Gender						
Male	-0.01(-0.02; 0.01)	0.291	Stationary	-0.01 (-0.03; 0.02)	0.634	Stationary
Female	-0.02 (-0.03; -0.01)	0.003	Decreasing	Not assessed	Not assessed	Not assessed
**Age group** (years)						
0-9	-0.01 (-0.05; 0.03)	0.637	Stationary	Not assessed	Not assessed	Not assessed
10-19	-0.01 (-0.03; 0.02)	0.492	Stationary	Not assessed	Not assessed	Not assessed
20-39	-0.01 (-0.02; 0.01)	0.390	Stationary	-0.01 (-0.03; 0.02)	0.608	Stationary
40-59	-0.02 (-0.03; -0.01)	0.008	Decreasing	0.01 (-0.02; 0.04)	0.632	Stationary
≥60	-0.01 (-0.04; 0.01)	0.172	Stationary	Not assessed	Not assessed	Not assessed
**Extremo Sul**	-0.01 (-0.02; 0.01)	0.492	Stationary	0.01 (-0.01; 0.01)	0.512	Stationary
Gender						
Male	-0.01 (-0.02; 0.01)	0.808	Stationary	0.01 (-0.02; 0.02)	0.946	Stationary
Female	-0.01 (-0.03; 0.01)	0.186	Stationary	Not assessed	Not assessed	Not assessed
**Age group** (years)						
0-9	-0.01 (-0.04; 0.02)	0.619	Stationary	Not assessed	Not assessed	Not assessed
10-19	0.01 (-0.03; 0.04)	0.655	Stationary	Not assessed	Not assessed	Not assessed
20-39	-0.01 (-0.02; -0.01)	0.003	Decreasing	Not assessed	Not assessed	Not assessed
40-59	0.01 (-0.01; 0.01)	0.829	Stationary	Not assessed	Not assessed	Not assessed
≥60	-0.01 (-0.03; 0.01)	0.347	Stationary	Not assessed	Not assessed	Not assessed

The incidence increased by 0.09%/year (95%CI 0.07; 0.17) in Jucuruçu, 0.03%/year (95%CI 0.01; 0.06) in Prado and 0.01%/year (95%CI 0.01; 0.03) in Santa Cruz Cabrália and reduced by 0.03%/year in Mucuri (95%CI -0.05; -0.02) and Guaratinga (95%CI -0.05; -0.01) (Figure 1C, data not shown).

The incidence in males reduced by 0.03%/year (95%CI -0.05; -0.01) in Mucuri and increased by 0.10%/year (95%CI 0.01; 0.19) in Jucuruçu and 0.03%/year in Prado (95%CI 0.01; 0.06) and in Santa Cruz Cabrália (95%CI 0.01; 0.05). In females, there was a decrease of 0.07%/year (95%CI -0.14; -0.01) in Medeiros Neto, 0.06%/year (95%CI -0.10; -0.02) in Mucuri, 0.03%/year (95%CI -0.050; -0.01) in Eunápolis and 0.02%/year (95%CI -0.03; -0.01) in Teixeira de Freitas (Figures 1D and 1E, data not shown).

In the 10-19 age group, there was an increase in incidence of 0.04%/year (95%CI 0.01; 0.08) in Itamaraju and 0.03%/year (95%CI 0.01; 0.06) in Prado. From 20-39 years old, there was a decrease of 0.06%/year (95%CI -0.10; -0.01) in Nova Viçosa and 0.03%/year (95%CI -0.06; -0.01) in Mucuri and an increase of 0.08%/year (95%CI 0.03; 0.13) in Prado. From 40-59 years old, there was an increase of 0.07%/year in Alcobaça (95%CI 0.01; 0.14) and Caravelas (95%CI 0.01; 0.13) and of 0.03%/year (95%CI 0.01; 0.05) in Prado, while there was a decrease of 0.06%/year (95%CI -0.11; -0.01) in Mucuri and 0.02%/year in Teixeira de Freitas (95%CI -0.04; -0.01). For those aged 60 or over, there was a decrease of 0.05%/year (95%CI -0.10; -0.01) in Eunápolis and 0.02%/year (95%CI -0.03; -0.01) in Itamaraju and an increase of 0.02%/year (95%CI 0.01; 0.05) in Porto Seguro (Figures 1F, 1G, 1H, 1I and 1J, data not shown). In some municipalities, it was not possible to assess the trend in the time series of general incidence by sex and age group due to the excessive presence of “zero” observations.

Estimating the temporal trend of mortality was impossible for municipalities and for some sex and age categories in the territories due to the high number of “zero” observations. In the analyses that could be done, the trends were shown to be stationary (Table 2). It was highlighted that Itanhém and Medeiros Neto reported deaths from new cases only after the emergence of the disease caused by the new coronavirus (COVID-19) and that Alcobaça, Belmonte, Itabela and Porto Seguro recorded their highest mortality rates after the start of the pandemic. 

## Discussion

The incidence and mortality from pulmonary tuberculosis were stationary in the territories between 2010 and 2023. The decreasing incidence occurred in females and in the age groups 0-9, 40-59 and 60 years or older. The Costa do Descobrimento showed a decreasing incidence for females and for the age group of 40-59 years, and the Extremo Sul showed a decreasing incidence for 20-39 years. This study identified that the population residing on the Costa do Descobrimento, male and aged 10-39, would be the most affected by pulmonary tuberculosis.

The average incidence of tuberculosis in the territories was high, with the Costa do Descobrimento maintaining the general incidence, by sex and age group, above that calculated in the Extremo Sul. Furthermore, the trend was stationary. Compared to the incidence of tuberculosis from 2011 to 2019, the mean incidence of the territories was above that observed for Bahia and the neighboring states of Espírito Santo, Piauí, Goiás, Minas Gerais, Sergipe and Tocantins (14). 

A decreasing trend in incidence was observed between 2001 and 2019 in Brazil (4,5,14). During this period, Bahia showed a drop in incidence of 4.8%/year, surpassed only by Piauí, with a drop of 6.1%/year (4). A divergent result was observed, with increasing incidence for Brazil and stationary for the Northeast between 2010 and 2019 (15). When assessing only the temporal trend of pulmonary tuberculosis, as in this study, an increasing incidence was observed between 2015 and 2019 and a stationary incidence in 2020 for Brazil and the Northeast region, which contradicts the result obtained previously (16). 

The high incidence of tuberculosis and its stationarity can be justified by the socioeconomic vulnerability of the territories. As susceptibility to tuberculosis is influenced by social determinants, the scope and efficiency of public tuberculosis control policies may be compromised (5,17). Income reflects the proportion of school dropouts and delays in education, with tuberculosis affecting individuals with low levels of education, which influences understanding of the disease and the risks of not treating it, keeping the chain of transmission active (17). 

The geographical location of the territories is also a factor to be considered (5,14). In the context of evaluating the spatiotemporal distribution of tuberculosis in Brazil, a cluster of high incidence of the disease was identified between the territories investigated and the northeast of Minas Gerais and the north of Espírito Santo (5). This cluster was considered to be at high risk for illness, suggesting that, to reduce the incidence in Bahian territories, coordinated actions by the governments of Bahia, Espírito Santo and Minas Gerais would be necessary (5). 

The high incidence of tuberculosis highlighted the need to strengthen local tuberculosis programs, especially on the Costa do Descobrimento, which maintained high rates of the disease. The stationary trend signaled the need for strategies that enhance the tracking, diagnosis and treatment of tuberculosis, with prioritization in municipalities with an increasing incidence trend.

The territories showed a decreasing incidence of the disease for females and a stationary incidence for males. This pattern was observed on the Costa do Descobrimento and in Medeiros Neto, Eunápolis and Teixeira de Freitas. In males, the increased incidence was observed in Jucuruçu, Prado and Santa Cruz Cabrália. Unlike these results, in Imperatriz in the states of Maranhão and Santa Catarina, the trend for both sexes was decreasing, a result that only occurred in Mucuri (18,19). Such differences may be due to the specific characteristics of the population of each municipality and the commitment of municipal health departments to meet targets for tuberculosis control.

Biological, social, cultural, behavioral and occupational factors can help understand the reduction in the incidence of tuberculosis in women (20). Unlike women, men may establish more social contacts, spend more time in environments prone to contamination, engage in occupations that pose a risk of disease or be exposed to lifestyle-related risk factors, such as smoking and excessive alcohol consumption (3,20,21). Factors related to genetic regulation and recruitment of immune cells modulated by sex hormones and X chromosome genes may confer advantages for females, which reduces the multiplication of mycobacteria in women (20,21).

A reduction in the incidence of tuberculosis in children under 9 years of age was observed. In line with this result, the spatial distribution of tuberculosis in Brazil, between 2010 and 2021, found an area of low occurrence of the disease for children under 15 years of age in the south of Bahia (22). This finding may have been influenced by the maintenance of high BCG vaccine coverage rates (23). In Bahia, BCG coverage is above 90.0% for the three largest municipalities in the territories: Eunapolis, Porto Seguro and Teixeira de Freitas (24). 

This study observed the decreasing trend for the age groups of 40-59 and 60 years and older. This finding corroborates results found in Imperatriz, where there was a decreasing trend for the age groups 15-59 and 60 years or older (19). Increasing incidence of tuberculosis and decreasing temporal trends are expected, due to population aging (25). As the population over 60 years of age has increased by almost 50.0% in Bahia over the last 12 years, this demographic change may have influenced the significantly negative annual percentage variation observed in this group (11,25). Porto Seguro was the only municipality that showed an increasing incidence in the population over 60 years old. In this age group, latent tuberculosis screening actions are recommended, given the difficulty in diagnosing elderly people who have other respiratory diseases with similar symptoms (26).

The average mortality rate from pulmonary tuberculosis in new cases for the territories was below the values recorded for the Northeast between 1990 and 1998 and for Bahia in the periods 1990-2015 and 2011-2019 (6,14). In this study, only mortality in new cases of the pulmonary form of tuberculosis was evaluated, which made it difficult to compare with other studies that considered not only new cases, but also mortality from all forms of the disease (6,7,14,27,28). 

Mortality was stationary for all sex and age categories and is in accordance with a study conducted between 2011 and 2019 in Bahia (14). A similar result was observed in Paraná, where overall tuberculosis mortality and mortality by sex remained stationary from 1998 to 2012 (27). As the Northeast and Bahia showed a downward trend in the cure of tuberculosis, it can be inferred that there was a greater risk of dissemination and worsening of the disease in the territories, which leads to the maintenance of deaths (15). It is worth noting that COVID-19 has changed the dynamics of tuberculosis in recent years, but inequalities in access to diagnosis and treatment date back to before the pandemic and should be considered as reasons why there has been no drop in mortality in the territories (19).

Deaths from tuberculosis are indicators of inequality in access to health and highlight the difficulty patients have in bearing the indirect costs of treatment (28,29). Regarding the economic burden of tuberculosis, 48.0% of Brazilian families spent more than 20.0% of their annual income on diagnosis, treatment or incapacity to work, which reflects the catastrophic costs of the disease (30). Under these conditions, there are worse prognoses for tuberculosis and, therefore, broad actions that involve not only access to diagnosis and treatment, but also lead to social protection are needed. Such actions can contribute to combating tuberculosis and reducing mortality from the disease (17).

Some municipalities reported deaths from tuberculosis or recorded their highest mortality rates after the COVID-19 emergency. Predictions using mathematical models suggested that COVID-19 would lead to a global increase in tuberculosis mortality (1,2). It was predicted that deaths from the disease would reach the same level as in 2013, nullifying a decade of efforts to reduce mortality, with a greater impact on developing countries (1). The increase in deaths would be the result of interruptions in diagnosis, care and treatment and the increase of individuals with undetected and untreated tuberculosis during the pandemic (1). However, given the underreporting of people with active tuberculosis who die, even before COVID-19, reduced access to diagnostic methods resulted in a worsening of reports of tuberculosis deaths after the start of the pandemic, which may make it difficult to assess mortality after 2020 (19).

Among the limitations of this study, the COVID-19 pandemic stood out, which influenced incidence and mortality trends worldwide (1,15). Another limitation was due to the use of secondary data from Sinan, which may be affected by underreporting or ignored/incomplete data. Some variables that could provide a greater understanding of the epidemiology of tuberculosis, such as race/skin color, education and clinical variables, contained many blank/ignored observations, making it impossible to perform trend analyses. The low sample size for some trends, especially for mortality, was also a limitation of this study, as it made most analyses unfeasible due to excessive “zero” observations. There was a limitation on the use of regional data, which reduces the representativeness of the research findings.

This study contributed to the understanding of the dynamics of pulmonary tuberculosis in two territories in southern Bahia and aims to assist in the adaptation of strategies and goals to combat the disease in the region. The study may also be relevant in fulfilling the commitments to eradicate tuberculosis undertaken by the government of Bahia, which are established in the World Health Organization’s Global Strategy to End Tuberculosis and in the Brazilian Ministry of Health’s National Plan to End Tuberculosis as a Public Health concern.

This study found a stationary trend for the incidence and mortality from pulmonary tuberculosis and a decreasing trend in incidence for females and the age groups 0-9, 40-59 and 60 years or older from 2010 to 2023. Since tuberculosis is a neglected disease, its temporal patterns can be modified by social and governmental changes. Reducing tuberculosis incidence and mortality rates will only be possible with a multisectoral approach related to health, education, and social protection. The particularities of each affected territory and municipality and the catastrophic costs faced by families to control the disease must be taken into account. 

## Data Availability

The database and analysis codes used in this study are available at: https://data.scielo.org/dataset.xhtml?persistentId=doi%3A10.48331%2Fscielodata.DDH8H2&version=DRAFT.
