# Supplementary material for: Temporal trends in incidence and mortality from pulmonary tuberculosis: time series study, Sul da Bahia, 2010-2023
Source: Epidemiol Serv Saude. 2025 Aug 4;34:e20240778. doi: 10.1590/S2237-96222025v34e20240778.en (PMC12338994; doi:10.1590/S2237-96222025v34e20240778.en)
Supplement: Translation

# Translation: Tendência temporal da incidência e mortalidade por tuberculose pulmonar: estudo de séries temporais, Sul da Bahia, 2010-2023

**DOI:** 10.1590/S2237-96222025v34e20240778.pt

**Authors:** Marília Caixeta de Araujo, Renata Soares Passinho, Renan Sallazar Ferreira Pereira, Delio José Mora

**Affiliations:** 1 Universidade Federal do Sul da Bahia, Centro de Formação em Ciências da Saúde, Teixeira de Freitas, BA, Brasil; 2 Universidade Federal de São João del-Rei, Departamento de Enfermagem, Divinópolis, MG, Brasil

**Keywords:** Tuberculose Pulmonar, Monitoramento Epidemiológico, Controle de Doenças Transmissíveis, Sistemas de Informação em Saúde, Estudos de Séries Temporais

## Abstract

**Objetivo:**

Analisar a tendência temporal da incidência e mortalidade por tuberculose pulmonar no sul da Bahia.

**Métodos:**

estudo de séries temporais dos casos novos e óbitos por tuberculose pulmonar realizado na Costa do Descobrimento e no Extremo Sul da Bahia. O número de casos novos e óbitos por tuberculose pulmonar foi obtido no Sistema de Informação de Agravos de Notificação, entre 2010 e 2023, e utilizado para cálculo da incidência e mortalidade. A regressão de Prais-Winsten foi utilizada para calcular a variação percentual anual (VPA) e o intervalo de confiança de 95% (IC95%) e classificar as tendências.

**Resultados:**

Registraram-se 4.005 casos novos e 128 óbitos por tuberculose pulmonar. A incidência média foi 34,91 casos por 100 mil habitantes, e a mortalidade, 1,11 óbitos por 100 mil habitantes. Incidência e mortalidade apresentaram tendências estacionárias. Incidência decrescente ocorreu no sexo feminino (VPA -0,01; IC95% -0,02; -0,01) e nas faixas etárias de 0-9 anos (VPA -0,02; IC95% -0,04; -0,01), 40-59 anos (VPA -0,01; IC95% -0,02; -0,01) e 60 anos ou mais (VPA -0,01; IC95% -0,04; -0,01). A Costa do Descobrimento apresentou incidência decrescente para o sexo feminino (VPA -0,02; IC95% -0,03; -0,01) e a faixa etária de 40-59 anos (VPA -0,02; IC95% -0,03; -0,01). No Extremo Sul, a incidência foi decrescente na faixa etária de 20-39 anos (VPA -0,01; IC95% -0,02; -0,01).

**Conclusão:**

Embora a tendência temporal da incidência e mortalidade tenham sido estacionárias, estratégias de saúde pública são necessárias, especialmente na Costa do Descobrimento, que manteve as maiores taxas da tuberculose pulmonar.

**Aspectos éticos**

Esta pesquisa utilizou bancos de dados de domínio público e anonimizados.:

## Introdução

A tuberculose é doença negligenciada que afeta principalmente populações de maior vulnerabilidade socioeconômica (1). Estima-se que um terço da população mundial esteja infectada pela *Mycobacterium tuberculosis* e que anualmente a doença seja responsável pelo adoecimento de 7,5 milhões de pessoas e por 1,3 milhão de mortes (1,2). Em 2023, o Brasil registrou 80.012 casos novos de tuberculose, dos quais 88,0% foram da forma pulmonar, com incidência de 32,75 casos pulmonares/100 mil habitantes (3). 

A queda da incidência por tuberculose no Brasil para todas as faixas etárias foi observada de 2001 a 2017 (4,5). As taxas de incidência por tuberculose foram maiores no sexo masculino, exceto na faixa etária de 10-14 anos (3). A tendência decrescente de óbitos no país foi observada entre 1990 e 2015, e indivíduos abaixo de 15 anos de idade registraram redução da mortalidade entre 1996 e 2020, com a maior queda para a faixa etária de 0-4 anos (6,7).

No Brasil, as desigualdades sociais refletem a heterogeneidade nos indicadores da tuberculose (4-6). Em 2023, a Bahia apresentou incidência de 25,90 casos/100 mil habitantes e o pior desempenho do Nordeste em casos curados (3). Entre 2001 e 2017, a Bahia registrou queda da incidência por tuberculose para todas as faixas etárias e, entre 1990 e 2015, a tendência de óbitos no estado foi decrescente (3,6,7). 

O Sistema Único de Saúde disponibiliza acesso ao diagnóstico e tratamento para a tuberculose no território nacional, mas essas ações ocorrem de forma desigual (8). O acesso aos serviços de saúde está relacionado às condições sociais e de residência dos indivíduos. Isso significa que pacientes com tuberculose vivendo em regiões com menor desenvolvimento socioeconômico enfrentam dificuldades, o que impacta na incidência e mortalidade por tuberculose pulmonar (9). 

A identificação de populações mais expostas à tuberculose permite o planejamento de ações de prevenção e controle em áreas de maior risco (10). Como há escassez de estudos sobre a tuberculose em determinadas regiões da Bahia e séries temporais podem fornecer visão longitudinal e dinâmica da saúde das populações (10), a análise do ponto de vista de sexo e faixa etária é relevante para avaliar quais grupos seriam mais afetados pela tuberculose. Este estudo buscou analisar a tendência temporal da incidência e mortalidade por tuberculose pulmonar em dois territórios do sul da Bahia. 

## Métodos

### Delineamento

Tratou-se de estudo de séries temporais que analizou a incidência e a mortalidade por tuberculose pulmonar no sul da Bahia no período 2010-2023.

### Contexto

Os territórios Costa do Descobrimento e Extremo Sul estão localizados no sul da Bahia (Figura 1A e 1B). Juntos, apresentam população de 824.903 habitantes, o que representa 5,8% da população baiana. 

**Figura 1:** Territórios Costa do Descobrimento e Extremo Sul da Bahia e classificação das tendências para as taxas de incidência por tuberculose pulmonar em seus municípios, 2010-2023 (n=4.005)

### Participantes

A população do estudo foi composta de indivíduos incluídos no Sistema de Informação de Agravos de Notificação (Sinan) como caso confirmado de tuberculose, com a forma clínica “pulmonar” e o tipo de entrada “caso novo”. Casos novos foram as pessoas com tuberculose que não usaram a medicação antituberculosa ou a usaram por menos de um mês (3). 

### Variáveis

Os dados selecionados foram: ano de notificação (2010-2023), sexo (masculino, feminino) e faixa etária em anos (0-9, 10-19, 20-39, 40-59 e 60 ou mais). Para a análise da mortalidade, utilizou-se o número de casos novos com situação de encerramento “óbito por tuberculose”. 

No cálculo das taxas de incidência e mortalidade por tuberculose pulmonar, foram utilizados os Censos Demográficos de 2010 e 2022 e as estimativas populacionais intercensitárias, disponibilizados pelo Instituto Brasileiro de Geografia e Estatística (11,12). O número de casos novos e de óbitos de casos novos foi dividido pela população residente e multiplicado por 100 mil habitantes. A população estimada para cada categoria de sexo e faixa etária foi usada para o cálculo das taxas de incidência e mortalidade nos territórios e seus municípios.

### Fontes de dados e mensuração

Os dados foram obtidos no Sinan, disponibilizados pelo Departamento de Informação e Informática do Sistema Único de Saúde e acessados via Tabnet em março de 2024 (13). O caminho para acesso aos dados foi: Acesso à informação > Informações de saúde (Tabnet) > Epidemiológicas e morbidade > Casos de tuberculose – desde 2001 > Abrangência geográfica Bahia. Em seguida, foram selecionados a linha “ano de diagnóstico”, a coluna “sexo” ou “faixa etária” e o conteúdo “casos confirmados”. Na aba “seleções disponíveis”, seção “Município de notificação”, foi sinalizado cada um dos 21 municípios que compõem os territórios de interesse. Em “seleções disponíveis”, foram sinalizadas as opções “caso novo” em “Tipo de entrada” e “pulmonar” em “Forma”. Para a análise da mortalidade, foi sinalizado “óbito por tuberculose” em “Situação de encerramento”.

Os dados da população dos municípios foram disponibilizados pelo Instituto Brasileiro de Geografia e Estatística e acessados em março de 2024 via Secretaria da Saúde da Bahia (11-13). O caminho para acesso aos dados foi: Vigilância em saúde > Vigilância epidemiológica > Demografia > População residente > Estratificada por sexo e faixa etária. Em seguida, foram selecionados a linha “ano”, a coluna “sexo” ou “faixa etária” e o conteúdo “população residente”. Na aba “seleções disponíveis”, foram sinalizados os 21 municípios investigados na seção “Município”.

### Controle de viés

Os dados estavam correlacionados em séries temporais, o que significa que o valor em um ponto no tempo estava relacionado com valores em pontos anteriores. Para a correção desse viés, foi aplicada a técnica de Prais-Winsten (10). 

O viés ecológico foi controlado ao não se assumir que associações observadas em nível agregado possam ser aplicadas individualmente, o que gera conclusões incorretas sobre relações causais. 

O viés de informação estava relacionado à qualidade e precisão dos dados secundários, uma vez que dados incompletos, imprecisos ou mal documentados podem distorcer resultados. Esse viés foi controlado ao selecionar as variáveis “sexo” e “faixa etária” que não possuíam a categoria “ignorado/em branco”.

### Métodos estatísticos

Os dados foram apresentados por meio de medidas de tendência central e dispersão para as variáveis quantitativas e de frequência para as qualitativas.

A regressão de Prais-Winsten foi empregada para a análise das séries temporais das taxas de incidência e mortalidade. Na análise, o tempo (ano) foi considerado variável independente, e as taxas de incidência e mortalidade, dependentes. A transformação logarítmica das taxas mencionadas foi realizada para reduzir o efeito da heterogeneidade dos dados, corrigir desvios de normalidade e estabilizar a variância no tempo (10).

Com base na regressão, estimaram-se a variação percentual anual e os intervalos de confiança de 95% (IC95%). A tendência foi: crescente, se p-valor<0,05 e variação percentual anual positiva; decrescente, se p-valor<0,05 e variação percentual anual negativa; e estacionária, se p-valor≥0,05. As análises foram realizadas no software STATA versão 14.0 (StataCorp LLC, College Station, TX, USA).

## Resultados

No período 2010-2023, foram notificados 4.005 casos novos de tuberculose pulmonar, com incidência média de 34,91 (±5,^10^) casos/100 mil habitantes para os territórios. A distribuição da incidência identificou picos de casos novos em 2011, 2014, 2019 e 2022 e declínios em 2017 e 2020 (Figura 2A). O maior número de casos novos foi registrado no sexo masculino (67,0%) e na faixa etária de 20-39 anos (43,0%). 

**Figura 2:** Série temporal da taxa de incidência (A) e da taxa de mortalidade (B) por tuberculose pulmonar. Costa do Descobrimento e Extremo Sul da Bahia, 2010-2023 (n=4.005)

Durante o período de estudo, foram observados 128 encerramentos de casos novos por óbito por tuberculose pulmonar. O maior número de óbitos ocorreu no sexo masculino (72,0%) e na população de 40-59 anos (37,0%). A média global foi 1,11 (±0,44) óbitos/100 mil habitantes (Figura 2B).

Observou-se a distribuição das taxas de incidência e mortalidade, segundo sexo e faixa etária (Tabela 1). A incidência média foi maior no sexo masculino e na faixa etária de 40-59 anos em ambos os territórios. A mortalidade média foi maior no sexo masculino e na faixa etária de 60 anos ou mais.

**Tabela 1:** Distribuição das taxas de incidência e mortalidade de tuberculose pulmonar, segundo sexo e faixa etária. Costa do Descobrimento e Extremo Sul da Bahia, 2010-2023 (n=4.005)

Variável	2010	2011	2012	2013		2014	2015	2016	2017		2018	2019	2020	2021	2022	2023	Média (desvio-padrão)
Taxa de incidência (por 100 mil habitantes)																	
Sexo																	
Masculino	54,28	54,29	38,35		47,57	54,57	42,48	39,09		37,88	43,35	53,31	38,55	46,14	59,97	47,68	46,96 (±7,33)
Feminino	25,34	27,19	26,68		26,72	31,53	22,67	19,89		16,69	20,34	24,75	19,87	19,03	22,25	16,98	22,85 (±4,34)
**Faixa etária** (anos)																	
0-9	7,24	3,58	4,97		4,66	6,60	6,54	3,25		3,87	3,32	2,64	3,04	1,51	4,10	3,28	4,19 (±1,65)
10-19	15,78	14,33	11,62		15,14	15,61	10,71	8,86		13,50	9,07	13,81	16,40	10,62	19,07	18,31	13,77 (±3,23)
20-39	53,81	54,45	39,56		52,36	52,59	44,63	37,53		31,98	43,04	56,35	37,73	38,91	53,17	41,67	45,56 (±7,99)
40-59	60,29	69,69	58,54		51,83	85,03	50,96	51,17		44,85	56,06	63,00	40,18	49,64	55,15	46,04	55,89 (±11,38)
≥60	63,22	61,27	52,13		63,93	51,30	49,60	51,94		50,10	46,28	52,57	36,45	53,34	52,11	32,34	51,19 (±8,85)
Taxa de mortalidade (por 100 mil habitantes)																	
Sexo																	
Masculino	2,36	2,08	0,51		2,41	1,91	1,42	1,18		1,64	1,45	1,91	1,44	1,66	1,47	0,98	1,60 (±0,52)
Feminino	0,79	0,78	0,00		0,49	1,44	1,67	0,47		0,00	0,00	0,48	0,47	0,70	1,44	0,00	0,62 (±0,56)
**Faixa etária** (anos)																	
0-9	0,72	0,00	0,00		0,00	0,00	0,65	0,00		0,65	0,66	0,00	0,00	0,00	0,00	0,00	0,19 (±0,32)
10-19	0,00	0,00	0,00		0,00	0,60	0,00	0,59		0,00	0,00	0,00	0,00	0,00	0,00	0,76	0,14 (±0,28)
20-39	0,79	1,57	0,78		1,82	0,72	1,79	0,00		0,35	0,36	0,72	0,36	1,44	1,19	0,00	0,85 (±0,62)
40-59	2,68	1,99	0,00		1,85	3,67	1,82	1,20		1,79	2,46	1,84	3,09	1,02	3,36	0,48	1,95 (±1,06)
≥60	7,35	5,84	0,00		5,44	6,75	5,36	5,33		2,64	0,00	6,74	1,01	4,03	1,80	1,80	3,86 (±2,60)

Na Costa do Descobrimento, a incidência média foi 34,93 (±7,81) casos/100 mil habitantes, e, no Extremo Sul, 26,73 (±4,62) casos/100 mil habitantes. A incidência de tuberculose pulmonar foi maior na Costa do Descobrimento para ambos os sexos e todas as faixas etárias quando comparada ao Extremo Sul. Na Costa do Descobrimento, a mortalidade média foi 1,25 (±0,80) óbitos/100 mil habitantes, e, no Extremo Sul, 0,53 (±0,^30^) óbitos/100 mil habitantes. No sexo feminino, a mortalidade média foi igual em ambos os territórios, enquanto, no sexo masculino, foi maior na Costa do Descobrimento. Para a faixa etária de 0-9 anos, verificou-se a mesma taxa de mortalidade média para os territórios. Para as demais faixas etárias, a mortalidade foi maior na Costa do Descobrimento. Ambos os territórios apresentaram a maior mortalidade média para a faixa etária de 60 anos ou mais (Tabela 1). 

A incidência de tuberculose pulmonar em ambos os territórios se mostrou estacionária, com queda para o sexo feminino e as faixas etárias de 0-9, 40-59 e 60 anos ou mais. Na Costa do Descobrimento, a incidência reduziu para o sexo feminino e para a faixa etária de 40-59 anos. No Extremo Sul, a incidência apresentou queda para a faixa etária de 20-39 anos (Tabela 2). 

**Tabela 2:** Variação percentual anual (VPA), intervalo de confiança de 95% (IC95%) e interpretação das tendências temporais da incidência e mortalidade por tuberculose pulmonar geral, segundo sexo e faixa etária. Costa do Descobrimento e Extremo Sul da Bahia, 2010-2023 (n=4.005)

	Taxa de incidência			Taxa de mortalidade		
Localidade	VPA (IC95%)	p-valor	Interpretação	VPA (IC95%)	p-valor	Interpretação
Territórios	-0,01 (-0,01; 0,01)	0,278	Estacionária	-0,01 (-0,02; 0,01)	0,423	Estacionária
Sexo						
Masculino	-0,01 (-0,01; 0,01)	0,912	Estacionária	-0,01 (-0,01; 0,01)	0,332	Estacionária
Feminino	-0,01 (-0,02; -0,01)	0,013	Decrescente	Não avaliado	Não avaliado	Não avaliado
**Faixa etária** (anos)						
0-9	-0,02 (-0,04; -0,01)	0,007	Decrescente	Não avaliado	Não avaliado	Não avaliado
10-19	0,01 (-0,01; 0,02)	0,594	Estacionária	Não avaliado	Não avaliado	Não avaliado
20-39	-0,01 (-0,02; 0,01)	0,295	Estacionária	-0,01 (-0,03; 0,01)	0,154	Estacionária
40-59	-0,01 (-0,01; -0,01)	0,014	Decrescente	0,01 (-0,02; 0,02)	0,762	Estacionária
≥60	-0,01 (-0,02; -0,01)	<0,001	Decrescente	-0,02 (-0,06; 0,01)	0,214	Estacionária
**Costa do Descobrimento**	-0,01 (-0,02; 0,01)	0,050	Estacionária	-0,01 (-0,02; 0,01)	0,650	Estacionária
Sexo						
Masculino	-0,01(-0,02; 0,01)	0,291	Estacionária	-0,01 (-0,03; 0,02)	0,634	Estacionária
Feminino	-0,02 (-0,03; -0,01)	0,003	Decrescente	Não avaliado	Não avaliado	Não avaliado
**Faixa etária** (anos)						
0-9	-0,01 (-0,05; 0,03)	0,637	Estacionária	Não avaliado	Não avaliado	Não avaliado
10-19	-0,01 (-0,03; 0,02)	0,492	Estacionária	Não avaliado	Não avaliado	Não avaliado
20-39	-0,01 (-0,02; 0,01)	0,390	Estacionária	-0,01 (-0,03; 0,02)	0,608	Estacionária
40-59	-0,02 (-0,03; -0,01)	0,008	Decrescente	0,01 (-0,02; 0,04)	0,632	Estacionária
≥60	-0,01 (-0,04; 0,01)	0,172	Estacionária	Não avaliado	Não avaliado	Não avaliado
**Extremo Sul**	-0,01 (-0,02; 0,01)	0,492	Estacionária	0,01 (-0,01; 0,01)	0,512	Estacionária
Sexo						
Masculino	-0,01 (-0,02; 0,01)	0,808	Estacionária	0,01 (-0,02; 0,02)	0,946	Estacionária
Feminino	-0,01 (-0,03; 0,01)	0,186	Estacionária	Não avaliado	Não avaliado	Não avaliado
**Faixa etária** (anos)						
0-9	-0,01 (-0,04; 0,02)	0,619	Estacionária	Não avaliado	Não avaliado	Não avaliado
10-19	0,01 (-0,03; 0,04)	0,655	Estacionária	Não Avaliado	Não avaliado	Não avaliado
20-39	-0,01 (-0,02; -0,01)	0,003	Decrescente	Não avaliado	Não avaliado	Não avaliado
40-59	0,01 (-0,01; 0,01)	0,829	Estacionária	Não avaliado	Não avaliado	Não avaliado
≥60	-0,01 (-0,03; 0,01)	0,347	Estacionária	Não avaliado	Não avaliado	Não avaliado

A incidência aumentou 0,09%/ano (IC95% 0,07; 0,^17^) em Jucuruçu, 0,03%/ano (IC95% 0,01; 0,06) em Prado e 0,01%/ano (IC95% 0,01; 0,03) em Santa Cruz Cabrália e reduziu 0,03%/ano em Mucuri (IC95% -0,05; -0,02) e Guaratinga (IC95% -0,05; -0,01) (Figura 1C, dados não apresentados).

A incidência no sexo masculino reduziu 0,03%/ano (IC95% -0,05; -0,01) em Mucuri e aumentou 0,10%/ano (IC95% 0,01; 0,^19^) em Jucuruçu e 0,03%/ano em Prado (IC95% 0,01; 0,06) e em Santa Cruz Cabrália (IC95% 0,01; 0,05). No sexo feminino, houve queda de 0,07%/ano (IC95% -0,14; -0,01) em Medeiros Neto, 0,06%/ano (IC95% -0,10; -0,02) em Mucuri, 0,03%/ano (IC95% -0,050; -0,01) em Eunápolis e 0,02%/ano (IC95% -0,03; -0,01) em Teixeira de Freitas (Figuras 1D e 1E, dados não apresentados).

Na faixa etária de 10-19 anos, houve aumento na incidência de 0,04%/ano (IC95% 0,01;0,08) em Itamaraju e 0,03%/ano (IC95% 0,01; 0,06) em Prado. De 20-39 anos, houve queda de 0,06%/ano (IC95% -0,10; -0,01) em Nova Viçosa e 0,03%/ano (IC95% -0,06; -0,01) em Mucuri e aumento de 0,08%/ano (IC95% 0,03; 0,^13^) em Prado. De 40-59 anos, verificou-se aumento de 0,07%/ano em Alcobaça (IC95% 0,01; 0,^14^) e Caravelas (IC95% 0,01; 0,^13^) e de 0,03%/ano (IC95% 0,01; 0,05) em Prado, enquanto houve queda de 0,06%/ano (IC95% -0,11; -0,01) em Mucuri e 0,02%/ano em Teixeira de Freitas (IC95% -0,04; -0,01). Para 60 anos ou mais, houve queda de 0,05%/ano (IC95% -0,10; -0,01) em Eunápolis e 0,02%/ano (IC95% -0,03; -0,01) em Itamaraju e aumento de 0,02%/ano (IC95% 0,01; 0,05) em Porto Seguro (Figuras 1F, 1G, 1H, 1I e 1J, dados não apresentados). Em alguns municípios, não foi possível avaliar a tendência das séries temporais da incidência geral por sexo e faixa etária devido à presença excessiva de observações “zero”.

A estimativa da tendência temporal da mortalidade foi impossibilitada para os municípios e algumas categorias do sexo e da faixa etária nos territórios devido ao elevado número de observações “zero”. Nas análises possíveis, as tendências mostraram-se estacionárias (Tabela 2). Ressaltou-se que Itanhém e Medeiros Neto notificaram óbitos de casos novos apenas após a emergência da doença pelo novo coronavírus (covid-19) e que Alcobaça, Belmonte, Itabela e Porto Seguro registraram suas maiores taxas de mortalidade após o início pandêmico. 

## Discussão

A incidência e a mortalidade por tuberculose pulmonar foram estacionárias nos territórios entre 2010 e 2023. A incidência decrescente ocorreu no sexo feminino e nas faixas etárias de 0-9, 40-59 e 60 anos ou mais. A Costa do Descobrimento apresentou incidência decrescente para o sexo feminino e a faixa etária de 40-59 anos, e o Extremo Sul, decrescente de 20-39 anos. Este estudo identificou que a população residente na Costa do Descobrimento, do sexo masculino e de 10-39 anos de idade seria a mais acometida pela tuberculose pulmonar.

A incidência média da tuberculose nos territórios mostrou-se elevada, com a Costa do Descobrimento mantendo a incidência geral, por sexo e faixa etária acima da calculada no Extremo Sul. Além disso, a tendência foi estacionária. Em comparação com a incidência de tuberculose de 2011 a 2019, a incidência média dos territórios encontrou-se acima da verificada para a Bahia e os estados vizinhos Espírito Santo, Piauí, Goiás, Minas Gerais, Sergipe e Tocantins (14). 

A tendência decrescente da incidência foi observada entre 2001 e 2019 no Brasil (4,5,14). Nesse período, a Bahia apresentou queda na incidência de 4,8%/ano, superada apenas pelo Piauí, com queda de 6,1%/ano (4). Observou-se resultado divergente, com incidência crescente para o Brasil e estacionária para o Nordeste entre 2010 e 2019 (15). Na avaliação apenas da tendência temporal da tuberculose pulmonar, como neste estudo, observou-se a incidência crescente entre 2015 e 2019 e estacionária em 2020 para o Brasil e a região Nordeste, o que contraria o resultado obtido anteriormente (16). 

A incidência elevada da tuberculose e sua estacionariedade podem ser justificadas pela vulnerabilidade socioeconômica dos territórios. Como a suscetibilidade à tuberculose é influenciada por determinantes sociais, o alcance e a eficiência das políticas públicas de controle da tuberculose podem estar comprometidos (5,17). A renda reflete a proporção de abandono e atraso escolar, com a tuberculose atingindo indivíduos com baixa escolaridade, o que influencia a compreensão da doença e dos riscos do não tratamento, o que mantém ativa a cadeia de transmissão (17). 

A localização geográfica dos territórios também é fator a ser considerado (5,14). No contexto de avaliar a distribuição espaço-temporal da tuberculose no Brasil, identificou-se aglomerado de alta incidência da doença entre os territórios investigados e o nordeste de Minas Gerais e o norte do Espírito Santo (5). Esse aglomerado foi considerado de alto risco para o adoecimento, sugerindo que, para a redução da incidência nos territórios baianos, seriam necessárias ações coordenadas dos governos da Bahia, do Espírito Santo e de Minas Gerais (5). 

A incidência elevada da tuberculose evidenciou a necessidade do fortalecimento dos programas locais de tuberculose, especialmente na Costa do Descobrimento, que manteve altas taxas da doença. A tendência estacionária sinalizou a necessidade de estratégias que potencializem o rastreamento, o diagnóstico e o tratamento da tuberculose, com priorização nos municípios com tendência crescente da incidência.

Os territórios apresentaram incidência decrescente da doença para o sexo feminino e estacionária para o masculino. Esse padrão foi observado na Costa do Descobrimento e em Medeiros Neto, Eunápolis e Teixeira de Freitas. No sexo masculino, o aumento da incidência foi observado em Jucuruçu, Prado e Santa Cruz Cabrália. Diferentemente desses resultados, em Imperatriz no Maranhão e em Santa Catarina, a tendência para ambos os sexos foi decrescente, resultado ocorrido apenas em Mucuri (18,19). Tais diferenças podem ser devidas às características próprias da população de cada município e ao empenho das secretarias municipais de saúde em cumprir metas para controle da tuberculose.

Fatores biológicos, sociais, culturais, comportamentais e ocupacionais podem auxiliar no entendimento da redução da incidência de tuberculose no sexo feminino (20). Diferentemente das mulheres, os homens podem estabelecer mais contatos sociais, passar mais tempo em ambientes propícios à contaminação, exercer ocupação de risco para a doença ou estar expostos a fatores de risco relacionados ao estilo de vida, como tabagismo e consumo excessivo de álcool (3,20,21). Fatores relacionados à regulação genética e ao recrutamento de células imunológicas modulados por hormônios sexuais e genes do cromossomo X podem conferir vantagens para o sexo feminino, o que reduz a multiplicação da micobactéria nas mulheres (20,21).

A redução da incidência de tuberculose para menores de 9 anos foi observada. Em concordância com esse resultado, a distribuição espacial da tuberculose no Brasil, entre 2010 e 2021, encontrou área de baixa ocorrência da doença para menores de 15 anos no sul da Bahia (22). Tal achado pode ter sido influenciado pela manutenção das taxas elevadas de cobertura da vacina BCG (23). Na Bahia, a cobertura da BCG está acima de 90,0% para os três maiores municípios dos territórios: Eunápolis, Porto Seguro e Teixeira de Freitas (24). 

Este estudo observou tendência decrescente para as faixas etárias de 40-59 e 60 anos ou mais. Esse achado corrobora resultados encontrados em Imperatriz, onde houve tendência decrescente para as faixas etárias de 15-59 e 60 anos ou mais (19). O aumento da incidência da tuberculose e as tendências temporais decrescentes são esperados devido ao envelhecimento populacional (25). Como nos últimos 12 anos a população acima de 60 anos aumentou quase 50,0% na Bahia, essa mudança demográfica pode ter influenciado a variação percentual anual significativamente negativa observada nesse grupo (11,25). Porto Seguro foi o único município que apresentou incidência crescente na população acima dos 60 anos. Nessa faixa etária, recomendam-se ações de rastreamento da tuberculose latente, visto a dificuldade diagnóstica em idosos que apresentam outras doenças respiratórias com sintomas semelhantes (26).

A taxa de mortalidade média por tuberculose pulmonar em casos novos para os territórios encontrou-se abaixo dos valores registrados para o Nordeste entre 1990 e 1998 e para a Bahia nos períodos 1990-2015 e 2011-2019 (6,14). Neste estudo, foi avaliada apenas a mortalidade em casos novos da forma pulmonar, o que dificultou a comparação com outras pesquisas que consideraram não somente os casos novos, mas também a mortalidade por todas as formas de entrada (6,7,14,27,28). 

A mortalidade mostrou-se estacionária para todas as categorias de sexo e faixa etária e está de acordo com estudo realizado entre 2011 e 2019 na Bahia (14). Resultado semelhante foi observado no Paraná, onde a mortalidade por tuberculose geral e por sexo manteve-se estacionária de 1998 a 2012 (27). Como o Nordeste e a Bahia apresentaram tendência de queda para a cura da tuberculose, pode-se inferir que houve maior risco de disseminação e agravamento da doença nos territórios, o que leva à manutenção dos óbitos (15). Cabe ressaltar que a covid-19 alterou a dinâmica da tuberculose nos últimos anos, mas as desigualdades de acesso ao diagnóstico e tratamento são advindas de tempos anteriores à pandemia e devem ser consideradas nas razões pelas quais não houve queda da mortalidade nos territórios (19).

As mortes por tuberculose são indicadores da desigualdade no acesso à saúde e evidenciam a dificuldade dos doentes em arcar com os custos indiretos do tratamento (28,29). Com relação à carga econômica da tuberculose, 48,0% das famílias brasileiras apresentaram gastos superiores a 20,0% de seus rendimentos anuais com diagnóstico, tratamento ou incapacidade para o trabalho, o que reflete os custos catastróficos da doença (30). Nessas condições, há piores prognósticos da tuberculose e, portanto, são necessárias ações amplas que envolvam não apenas o acesso ao diagnóstico e ao tratamento, mas também levem à proteção social. Tais ações podem contribuir para o enfrentamento da tuberculose e a redução da mortalidade pela doença (17).

Alguns municípios notificaram óbitos pela tuberculose ou registraram suas maiores taxas de mortalidade após a emergência da covid-19. Previsões utilizando modelos matemáticos sugeriram que a covid-19 levaria a um aumento global na mortalidade por tuberculose (1,2). Foi previsto que as mortes pela doença chegariam ao mesmo patamar de 2013, o que anularia uma década de esforços para a redução da mortalidade, com maior impacto nos países em desenvolvimento (1). O aumento dos óbitos seria efeito das interrupções no diagnóstico, nos cuidados e no tratamento; além da expansão dos indivíduos com tuberculose não detectada e não tratada durante a pandemia (1). Porém, dada a subnotificação de pessoas com tuberculose ativa que morrem, mesmo antes da covid-19, a redução do acesso aos meios diagnósticos resultou em piora das notificações das mortes por tuberculose após início da pandemia, o que pode dificultar a avaliação da mortalidade após 2020 (19).

Entre as limitações deste estudo, destacou-se a pandemia da covid-19, a qual influenciou as tendências de incidência e mortalidade em todo o mundo (1,15). Outra limitação se deveu à utilização de dados secundários do Sinan, que podem ser afetados por subnotificações ou por dados ignorados/incompletos. Algumas variáveis que poderiam proporcionar maior entendimento da epidemiologia da tuberculose, como raça/cor da pele, escolaridade e variáveis clínicas, continham muitas observações em branco/ignoradas, o que inviabilizou a realização das análises de tendências. O baixo número amostral para algumas tendências, principalmente para a mortalidade, também limitou o estudo ao inviabilizar as análises por excessivas observações “zero”. Houve a limitação da utilização de dados regionais, o que reduz a representatividade dos achados da pesquisa.

Este estudo trouxe contribuições para o entendimento da dinâmica da tuberculose pulmonar em dois territórios do sul da Bahia e pretende auxiliar na adaptação de estratégias e metas para o enfrentamento da doença na região. O estudo pode ainda ser relevante no cumprimento dos compromissos assumidos pelo governo da Bahia para a erradicação da tuberculose que estão estabelecidos na Estratégia Global para o Fim da Tuberculose da Organização Mundial de Saúde e no Plano Nacional pelo Fim da Tuberculose como Problema de Saúde Pública do Ministério da Saúde.

Este estudo encontrou tendência estacionária para a incidência e mortalidade por tuberculose pulmonar e tendência decrescente na incidência para o sexo feminino e as faixas etárias de 0-9, 40-59 e 60 anos ou mais de 2010 a 2023. Sendo a tuberculose uma doença negligenciada, seus padrões temporais podem ser modificados a partir de alterações sociais e governamentais. A redução das taxas de incidência e mortalidade pela tuberculose só será possível com abordagem multissetorial relacionada à saúde, educação e proteção social. Devem-se levar em consideração as particularidades de cada território e município afetado e os custos catastróficos enfrentados pelas famílias para o controle da doença. 

## Data Availability

O banco de dados e os códigos de análise utilizados na pesquisa estão disponíveis em: https://data.scielo.org/dataset.xhtml?persistentId=doi%3A10.48331%2Fscielodata.DDH8H2&version=DRAFT.
